# Biofilm Formed by *Candida haemulonii* Species Complex: Structural Analysis and Extracellular Matrix Composition

**DOI:** 10.3390/jof6020046

**Published:** 2020-04-03

**Authors:** Lívia S. Ramos, Thaís P. Mello, Marta H. Branquinha, André L. S. Santos

**Affiliations:** 1 Laboratório de Estudos Avançados de Microrganismos Emergentes e Resistentes (LEAMER), Departamento de Microbiologia Geral, Instituto de Microbiologia Paulo de Góes (IMPG), Universidade Federal do Rio de Janeiro (UFRJ), Rio de Janeiro 21941-901, Brazil; liviaramos2@yahoo.com.br (L.S.R.); thaispdmello@gmail.com (T.P.M.); mbranquinha@micro.ufrj.br (M.H.B.); 2 Programa de Pós-Graduação em Bioquímica (PPGBq), Instituto de Química (IQ), UFRJ, Rio de Janeiro 21941-909, Brazil

**Keywords:** *Candida haemulonii* complex, biofilm, extracellular matrix, catheter, polystyrene, virulence

## Abstract

*Candida haemulonii* species complex (*C. haemulonii*, *C. duobushaemulonii*, and *C. haemulonii* var. *vulnera*) has emerged as opportunistic, multidrug-resistant yeasts able to cause fungemia. Previously, we showed that *C. haemulonii* complex formed biofilm on polystyrene. Biofilm is a well-known virulence attribute of *Candida* spp. directly associated with drug resistance. In the present study, the architecture and the main extracellular matrix (ECM) components forming the biofilm over polystyrene were investigated in clinical isolates of the *C. haemulonii* complex. We also evaluated the ability of these fungi to form biofilm on catheters used in medical arena. The results revealed that all fungi formed biofilms on polystyrene after 48 h at 37 °C. Microscopic analyses demonstrated a dense network of yeasts forming the biofilm structure, with water channels and ECM. Regarding ECM, proteins and carbohydrates were the main components, followed by nucleic acids and sterols. Mature biofilms were also detected on late bladder (siliconized latex), nasoenteric (polyurethane), and nasogastric (polyvinyl chloride) catheters, with the biomasses being significantly greater than on polystyrene. Collectively, our results demonstrated the ability of the *C. haemulonii* species complex to form biofilm on different types of inert surfaces, which is an incontestable virulence attribute associated with devices-related candidemia in hospitalized individuals.

## 1. Introduction

*Candida haemulonii*, *Candida duobushaemulonii,* and *Candida haemulonii* var. *vulnera* form a fungal complex (named *C. haemulonii* complex) that is represented by emergent, opportunistic yeasts able to cause human infections with a wide range of clinical manifestations, varying from superficial to deep-seated infections, especially in individuals with immunocompromising health conditions [1]. In this sense, the main isolation sites of the *C. haemulonii* species complex described in the literature are blood, foot ulcers, nails, bones, skin wounds, and vagina; however, there are reports of isolates obtained from other body fluids such as cerebrospinal fluid, bronchoalveolar lavage, vaginal discharge, pleural effusion, peritoneal and ascitic fluids, bile, and urine [1,2,3,4,5,6,7,8,9,10,11,12,13,14,15].

The multidrug-resistance profile of the *C. haemulonii* species complex has been highlighted by many research groups worldwide, making it a challenge to treat such infections, which is aggravated by the immunological status of the majority of target patients. Although the knowledge about this fungal complex has been growing in recent years, many aspects related to its virulence need to be better investigated. In this sense, biofilm formation is an unquestionable and well-known virulence attribute associated with both bacterial and fungal infections around the world. Biofilm formation by the *C. haemulonii* species complex has already been reported based on the use of classical methodologies [1,7,16], but there is lack of information about the characteristics of the biofilm formed by these fungi. Indeed, it is believed that biofilm lifestyle is the preferred organization mode of microorganisms in nature, which is characterized by a highly complex structured community of microorganisms that interact with each other and with a biotic/abiotic surface, covered by a self-produced extracellular matrix (ECM) composed mainly of proteins, polysaccharides, lipids, nucleic acids, minerals, and water [17,18]. Functionally, the ECM plays an important role in the biofilm maintenance, architecture, and dynamic, being responsible for conferring protection against external stressors, such as host immune responses (both humoral and cellular components) and drugs (either disinfectants or antimicrobial agents), which directly impact the treatment, especially that of seriously ill patients [18,19].

Biofilm-related infections are considered a huge problem in healthcare settings worldwide [20]. Many chronic infections caused by both bacteria and fungi have been associated to biofilm mode of growth, including lung infections (e.g., fungal ball) and chronic leg wounds [20]. *Candida* species, for example, can form biofilm on a variety of medical devices, and it is well-known that catheter-related fungemia is associated with high morbidity and mortality rates among patients in healthcare services, despite the consequent financial burden related to this situation [20]. *C. haemulonii* complex has already been associated to cases of catheter-related fungemia in both pediatric and elderly patients [6,21], and the catheter, in this scenario, acts as a gateway to the infection development as well as to its chronicity.

In the present study, we aimed to investigate the architecture of the biofilm formed by 12 clinical isolates comprising the *C. haemulonii* species complex (*C. haemulonii*, *n* = 5; *C. duobushaemulonii*, *n* = 4; and *C. haemulonii* var. *vulnera*, *n* = 3) on polystyrene, with a special focus on the study of the chemical composition of their ECM. Additionally, we evaluated and compared the ability of these fungal isolates to form biofilm on different medical devices commonly applied in clinical settings, such as nasogastric, late bladder, and nasoenteric catheters made of polyvinyl chloride, siliconized latex, and polyurethane, respectively.

## 2. Materials and Methods

### 2.1. Microorganisms and Growth Conditions

A total of 12 clinical isolates recovered from patients from Brazilian hospitals between 2005 and 2013 and identified by molecular approaches as belonging to the *C. haemulonii* species complex were used in the present work [10]. Some relevant data about the fungal isolates are summarized in Table 1. Fungal cells were cultured in Sabouraud dextrose medium (under the following conditions: 37 °C for 48 h at 200 rpm) and then used in all the experiments. The yeast cells were quantified using a Neubauer chamber.

### 2.2. Biofilm Formation on Polystyrene

Fungal cell suspensions in Sabouraud broth (200 µL containing 10^6^ yeasts) were transferred into each well of a flat-bottom 96-well polystyrene microtiter plate, and then incubated without agitation at 37 °C for 48 h. Plate wells containing only culture medium were used to set up the reader as blanks. The supernatant fluids were removed by pipetting and, subsequently, the plate wells were washed three times with phosphate-buffered saline (PBS, pH 7.2) to remove nonadherent cells. The measurements of biofilm parameters (biomass, metabolic activity, and ECM) were then performed as described below.

### 2.3. Biofilm Parameters

#### 2.3.1. Biomass

Biomass quantification was performed as described by Peeters et al. [22]. Firstly, methanol at 99% (200 μL) was used to fix the biofilms for 15 min at room temperature, then the supernatant was discarded, and the plates were air-dried during 5 min. Afterwards, the plates were incubated for 20 min at room temperature with 0.4% crystal violet solution (200 μL; stock solution diluted in PBS; Sigma-Aldrich, St Louis, MO, USA). The plate wells were finally washed once with PBS in order to remove the excess of staining and the bound dye was then eluted with 33% acetic acid (200 μL) for 5 min. The acetic acid solution (100 μL) was transferred to a new 96-well plate and the absorbance was measured using a microplate reader at 590 nm (SpectraMax M3; Molecular Devices, Sunnyvale, CA, USA).

#### 2.3.2. Metabolic Activity

The metabolic activity of the biofilm was determined using a colorimetric assay able to measure the metabolic reduction of 2,3-bis (2-methoxy-4-nitro-5-sulfophenyl)-5-[(phenylamino) carbonyl]-2H-tetrazolium hydroxide (XTT; Sigma-Aldrich, St Louis, MO, USA) to a water-soluble brown formazan product [22]. The XTT/menadione solution was prepared by dissolving 2 mg XTT in 10 mL of pre-warmed PBS, which was supplemented with 100 μL of a stock solution of menadione (0.4 mM in acetone). The XTT/menadione solution (200 μL) was added to the plate wells and incubated at 37°C for 3 h in the dark. Afterwards, 100 μL of supernatant from each well was transferred to a new microplate and the colorimetric changes were quantified using a microplate reader at 492 nm (SpectraMax M3; Molecular Devices, San Jose, CA, USA).

#### 2.3.3. ECM

The biofilm ECM was quantified according to the method described by Choi et al. [23]. Briefly, 0.1% safranin (200 μL; Sigma-Aldrich, St Louis, MO, USA) diluted in PBS was used to stain the nonfixed biofilms, at room temperature for 5 min. Afterwards, the plate wells were washed once with PBS and the bound dye was eluted with 30% acetic acid (200 μL). Supernatants (100 μL) were transferred to a new 96-well plate and absorbance was quantified using a microplate reader at 530 nm (SpectraMax M3; Molecular Devices, San Jose, CA, USA).

### 2.4. Biofilm Architecture

#### 2.4.1. Confocal Laser Scanning Microscopy (CLSM)

Biofilms formed on polystyrene surface for 48 h at 37 °C were stained with 5 µg/mL of Calcofluor white (Sigma-Aldrich, San Luis, MO, USA) for 1 h at room temperature, protected from the light [24,25]. Subsequently, the biofilms were washed twice with PBS and covered with *n*-propylgallate for observation under a confocal microscope (Leica TCS SP5 with OBS, Berlin, Germany). Three-dimensional reconstitutions of biofilms were obtained by Fiji (ImageJ2, UW-Madison LOCI, Wisconsin, WI, USA) software [26]. The analysis of images was conducted using *z*-series image stacks from spots of each biofilm chosen randomly.

#### 2.4.2. Scanning Electron Microscopy (SEM)

Biofilms formed on polystyrene coverslips (Nalgene, Thermo Fisher Scientific, Waltham, MA, USA), at 37 °C for 48 h, were fixed in a solution made of 2.5% glutaraldehyde in 0.1 M sodium cacodylate buffer, pH 7.2, at 4 °C overnight. Then, PBS was used to wash the systems, which were post-fixed with 2% osmium tetroxide for 2 h. Dehydration was done in graded concentrations of acetone (25%–100%). The critical point method was used to dry fungal biofilms, which were then mounted on stubs, coated with gold (20–30 nm), and analyzed using a JEOL JSM 6490LV scanning electron microscope [27,28].

### 2.5. Biofilm ECM Composition

#### 2.5.1. Extraction of ECM

Biofilms formed on polystyrene for 48 h at 37 °C were washed three times with PBS to remove the medium and nonadherent cells. Then, 200 µL of 1.5 M NaCl was added to each well of the microtiter plate and incubated overnight at 4 °C [29]. Finally, the well contents were transferred to a clean tube and filtered through a 0.22-μm membrane (Millipore, São Paulo, SP, Brazil).

#### 2.5.2. Chemical Quantification of the Main Biomolecules

The protein concentration was determined by the method described by Lowry et al. [30], using bovine serum albumin (BSA; Sigma-Aldrich, San Luis, MO, USA) as standard. The carbohydrate concentration was determined by the method described by Dubois et al. [31], with some modifications. Briefly, the experiment was carried out using a polystyrene 96-well microplate, in which 50 µL of the extracellular matrix, 150 µL of sulfuric acid, and 30 µL of 80% phenol were added. The standard curve was made with glucose (Sigma-Aldrich, San Luis, MO, USA). The plate was heated in a water bath for 10 min at 90 °C, and then incubated at room temperature for 5 min. Finally, the absorbance was measured at 530 nm using a microplate reader (SpectraMax M3; Molecular Devices, San Jose, CA, USA). The nucleic acids present in ECM were extracted with the Gentra^®^ Puregene^®^ Yeast and G+ Bacteria Kit (Qiagen^®^, Maryland, MD, USA), according to the manufacturer’s protocol, and then quantified using a spectrophotometer (Nano-Vue PlusTM; GE Healthcare, Chicago, IL, USA). The sterol concentration was determined using the AmplexTM Red Cholesterol Assay kit (Thermo Fisher Scientific, Waltham, MA, USA), according to the manufacturer’s instructions.

### 2.6. Biofilm Formation on Distinct Catheters Employed in Clinical Settings

In order to evaluate the ability of *C. haemulonii* species complex to form biofilm on common medical devices, a nasogastric catheter composed by polyvinyl chloride (Medsonda, Arapoti, PR, Brazil), a late bladder catheter composed by siliconized latex (Sisco, São Paulo, SP, Brazil), and a nasoenteric catheter composed by polyurethane (Solumed, Atuba-Pinhais, PR, Brazil) were selected. Autoclaved scissors were used to cut catheters into pieces of approximately 0.30, 0.70, and 0.36 cm^2^, respectively, and placed on flat-bottom 96-well microplates. Fungal cell suspensions were placed on the catheters in flat-bottom 96-well plates (using polystyrene substratum as control) in Sabouraud medium (10^6^ yeasts in 200 µL) at 37 °C for 48 h. Blank controls were prepared by adding only culture medium to the catheters. Then, the catheters were washed three times with PBS to remove nonadherent cells and carefully transferred to a new flat-bottom 96-well microplate, and then the biofilm biomass was measured as described earlier.

### 2.7. Statistics

All experiments were performed in triplicate, in three independent experimental sets. The results were analyzed statistically by Student’s *t*-test (in the comparisons between two groups) and one-way analysis of variance (ANOVA) (in the comparisons between three or more groups). The correlation tests were determined by Pearson’s correlation coefficient (*r*). All analyses were performed using the program GraphPad Prism5. In all analyses, *p*-values of 0.05 or less were considered statistically significant.

## 3. Results and Discussion

### 3.1. Biofilm on Polystyrene Surface: Classical Parameters

**Figure 1:**
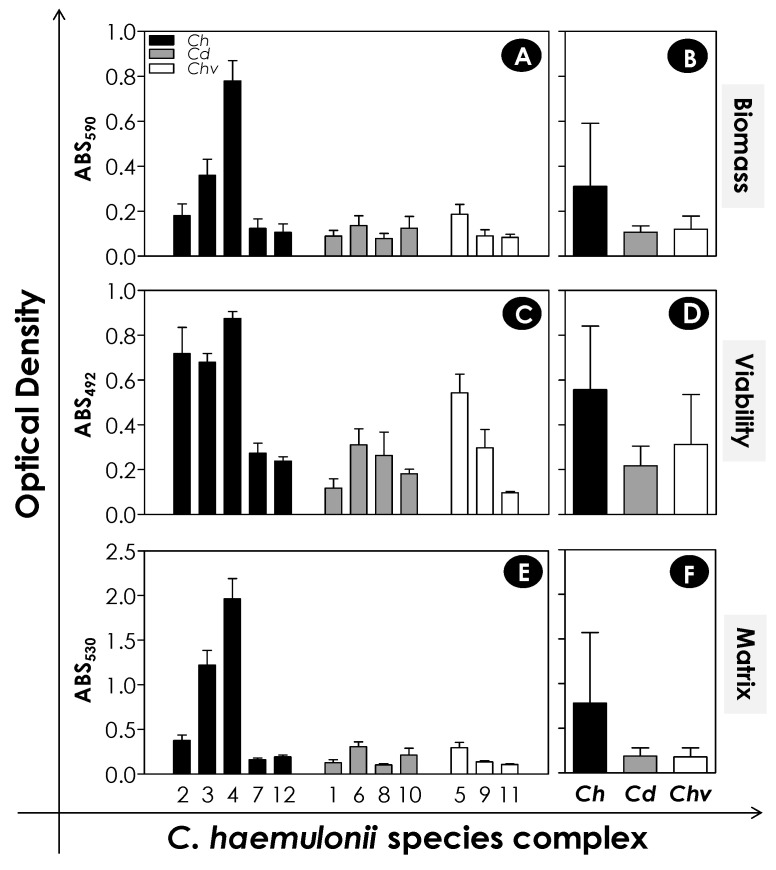
Biofilm formation by the *C. haemulonii* species complex on polystyrene surface. Yeasts (200 μL containing 10^6^ cells) were placed to interact with polystyrene for 48 h at 37 °C. Afterwards, the systems were processed to detect fungal biomass by crystal violet incorporation in methanol-fixed biofilms at 590 nm, cell viability by the reduction of 2,3-bis (2-methoxy-4-nitro-5-sulfophenyl)-5-[(phenylamino) carbonyl]-2H-tetrazolium hydroxide (XTT) in formazan at 492 nm, and extracellular matrix by staining non-fixed biofilms with safranin at 530 nm. The results were expressed as absorbance (ABS) values per clinical isolate studied (**A**,**C**,**E**) and mean per fungal species (**B**,**D**,**F**). The results are shown as mean ± standard deviation of three independent experiments. The numbers on the X-axis of graphs represent each of the 12 clinical isolates of the *C. haemulonii* species complex studied, in which *Ch* means *C. haemulonii*, *Cd* means *C. duobushaemulonii,* and *Chv* means *C. haemulonii* var. *vulnera*.

It is known that adhesion represents the first step for biofilm formation, which is an important virulence attribute described for several *Candida* species presenting medical implications [32,33]. The relevance of biofilm formation by *Candida* spp. lies the crucial characteristics such as greater resistance to antifungal drugs, host immune responses, and stress situations, resulting in difficulties in the treatment and possible persistence of the infectious process [17]. Taking this into consideration, initially, we confirmed the capacity of clinical isolates belonging to the *C. haemulonii* complex to form biofilm over a polystyrene surface [16]. In this set of experiments, three classical parameters related to biofilm formation were evaluated after 48 h of contact with polystyrene: (i) biomass by the incorporation of crystal violet in methanol-fixed cells, (ii) metabolic activity (cell viability) by reduction of XTT, and (iii) ECM by absorption of safranin, in the latter cases, using non-fixed fungal cells. All 12 clinical isolates comprising the *C. haemulonii* complex formed biofilm at different degrees, exhibiting a typical isolate-specific pattern (Figure 1A,C,E). Statistically significant differences were not observed, while the average measurements of the three biofilm parameters among the three fungal species forming the *C. haemulonii* complex were compared (Figure 1B,D,F). Biofilms revealed by the incorporation of crystal violet and safranin showed the presence of a network formed by yeasts and an exuberant ECM, respectively (data not shown).

Regarding the biofilm biomass, we observed that the average of biofilm formation on polystyrene by the clinical isolates studied herein was similar to that reported by Cendejas-Bueno et al. [1], who also studied clinical isolates of the *C. haemulonii* complex obtained from different isolation sites. The comparison of biofilm formation among the members of other *Candida* species complex has already been documented. In this sense, the three species of the *C. parapsilosis* complex (*C. parapsilosis* sensu strictu, *C. orthopsilosis*, and *C. metapsilosis*) exhibited similar abilities to produce mature biofilms on abiotic surfaces regarding biomass, viability, and three-dimensional architecture [34,35,36]. Regarding the *C. glabrata* complex, Figueiredo-Carvalho et al. [37] reported that biofilm biomass was significantly higher than *C. nivariensis*.

### 3.2. CLSM Analysis

**Figure 2:**
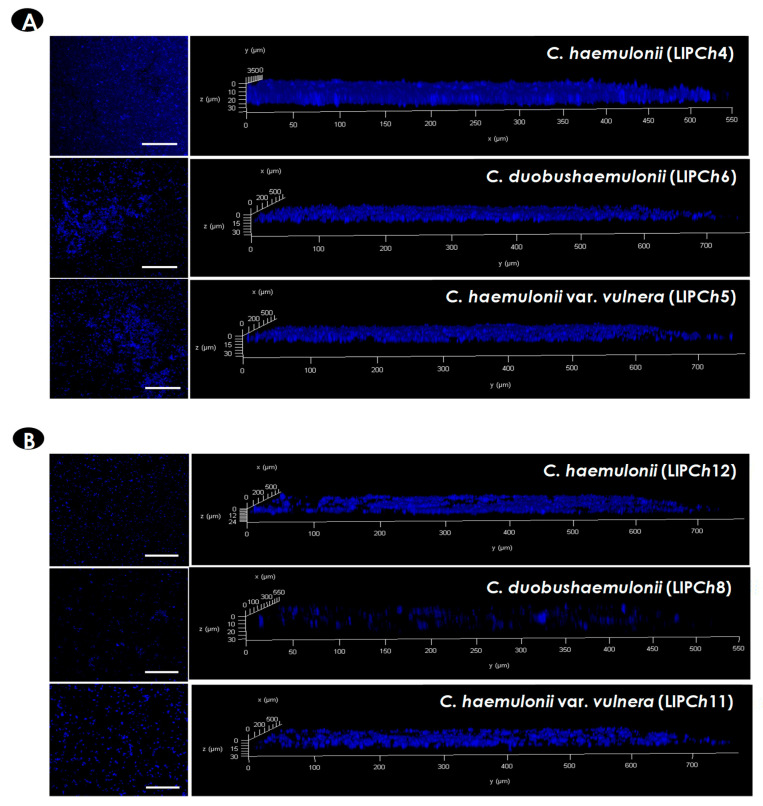
Representative confocal laser scanning microscopy (CLSM) images of the biofilms formed by the *C. haemulonii* species complex on polystyrene surface. Yeasts (200 μL containing 10^6^ cells) were placed to interact with polystyrene for 48 h at 37 °C. Subsequently, the biofilms were stained with Calcofluor white, evidencing the fungal biomass. The panels on the left represent the top view images of the fungal biofilms visualized by CLSM; bars represent 5 µm. The graphs on the right represent the three-dimensional reconstruction of the biofilms formed by each species. The isolates *C. haemulonii* (LIP*Ch*4), *C. duobushaemulonii* (LIP*Ch*6), and *C. haemulonii* var*. vulnera* (LIP*Ch*5), which formed the most robust biofilms (**A**), as well as the isolates *C. haemulonii* (LIP*Ch*12), *C. duobushaemulonii* (LIP*Ch*8), and *C. haemulonii* var. *vulnera* (LIP*Ch*11), which formed the weakest biofilms (**B**), are shown.

The three-dimensional organization as well as the biomass distribution in the biofilms formed by the clinical isolates comprising the *C. haemulonii* complex were analyzed by CLSM (Figure 2), which is a nondestructive technique that allows in situ visualization of the intact biofilm [38]. To do it, Calcofluor white was used to stain the yeasts owing to its affinity to chitin (which is a universal polysaccharide present in the fungal cell wall), evidencing the biofilm biomass as well as the ECM (Figure 2), which is evidenced by the diffuse marking between the yeasts, as previously proposed [39]. In addition, the three-dimensional representation of biofilms was used to determine their thickness, which ranged from 21.6 to 39.1 μm (overall mean = 28.3 ± 5.6 μm) for all clinical isolates studied. The biofilm thickness in each fungal species is as follows: *C. haemulonii*, 21.6 to 32.1 μm (overall mean = 26.1 ± 4.8 μm); *C. duobushaemulonii*, 25.9 to 39.1 μm (mean = 30.5 ± 5.8 μm); and *C. haemulonii* var. *vulnera*, 26.1 to 37.1 μm (mean = 29.1 ± 7.1 μm) (Figure 2). Some authors have documented different thicknesses of biofilms formed by *Candida* species, varying from 11 to 13 μm for *C. tropicalis* [40], 25 to 77 μm for *C. albicans* [39,41], 35.2 to 81.2 μm for *C. famata* [42], and 21 to 26 μm for *C. auris* [43]. In this sense, a variety of conditions can interfere with biofilm features, including isolate specificities, planktonic growth, initial inoculum concentration, and variability on biofilm-forming conditions (substratum, temperature, CO_2_ tension, fluid flow, developmental timing, and medium used to support biofilm formation) [44].

### 3.3. SEM Analysis

**Figure 3:**
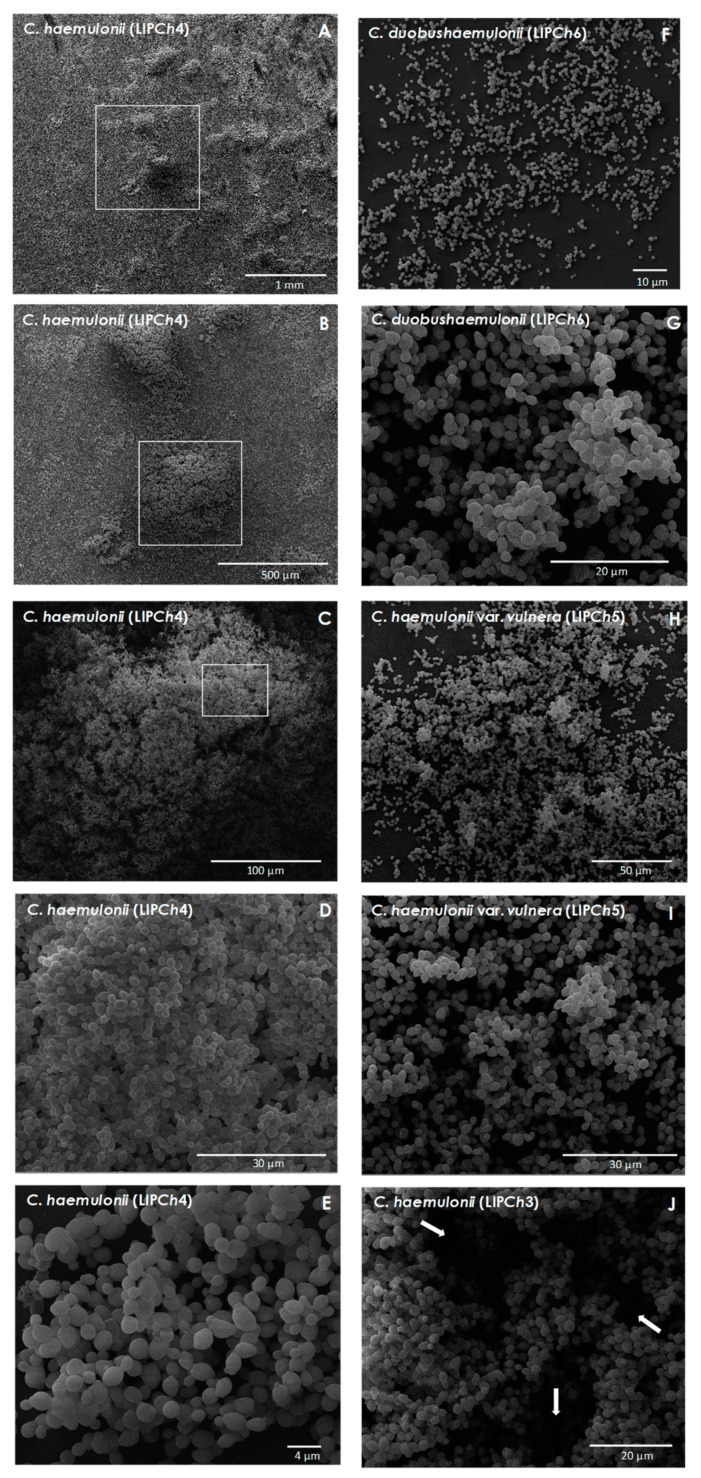
Representative low-magnification scanning electron microscopy (SEM) images of the biofilms formed by the *C. haemulonii* species complex on polystyrene surface. Yeasts (200 μL containing 10^6^ cells) were placed to interact with polystyrene coverslips for 48 h at 37 °C, after which the coverslips were visualized using SEM. The images revealed a dense network of yeast cells. In the panel, the images on the left side exhibit different magnifications of the biofilm formed by the isolate LIP*Ch*4 of *C. haemulonii* (**A**–**E**) while on the right side, it is possible to see the biofilms of isolate LIP*Ch*6 of *C. duobushaemulonii* (**F**,**G**) and LIP*Ch*5 of *C. haemulonii* var. *vulnera* (**H**,**I**). Representative water channels are indicated by white arrows in the image of isolate LIP*Ch*3 of *C. haemulonii* (**J**). Note that the white square in (**A**) is the place that was chosen to be amplified and shown in (**B**), and this logic sequence was used in the left side images from (**A**) to (**D**).

**Figure 4:**
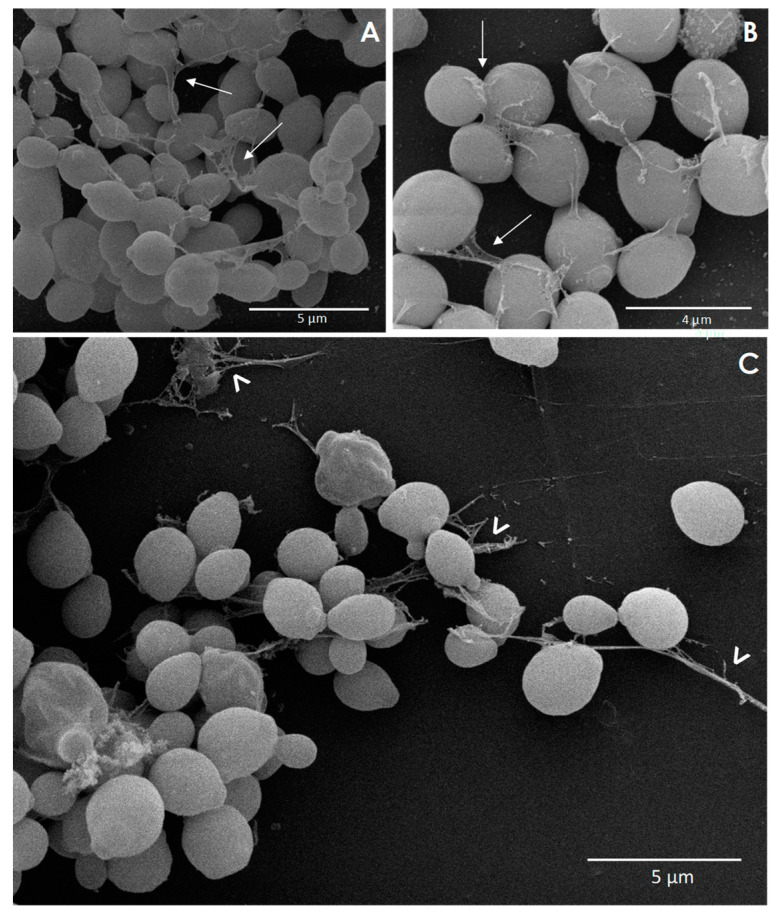
Representative high-magnification SEM images of the biofilms formed by the *C. haemulonii* species complex on polystyrene surface, focusing on the extracellular matrix (ECM). Yeasts (200 μL containing 10^6^ cells) were placed to interact with polystyrene coverslips for 48 h at 37 °C, after which the coverslips were visualized using SEM. The ECM of biofilms of *C. haemulonii* LIP*Ch*4 (**A**), *C. duobushaemulonii* LIP*Ch*6 (**B**), and *C. haemulonii* var. *vulnera* LIP*Ch*5 (**C**) are indicated by symbols. The images clearly reveal the presence of an ECM surrounding and holding the yeast cells together (white thin arrows) as well as connecting the yeasts with the polystyrene surface (white thick arrowheads).

SEM analysis was used to assess the biofilm ultrastructure and to evidence peculiar morphological characteristics. Mature biofilms of *C. haemulonii* species complex consisted of a dense network of yeast cells, while structures similar to pseudohyphae were scarcely observed in the majority of the isolates studied (Figure 3). As seen through other approaches, isolate-specific differences were also visualized in biofilm ultrastructure. In this sense, the biofilms formed by *C. haemulonii* isolates LIP*Ch*3 and LIP*Ch*4, for example, exhibited a continuous, intimately packed multilayer structure (Figure 3A–E), while in the remaining fungal isolates, the biofilms were formed by a predominantly discontinuous monolayer with cell aggregates (Figure 3G–I). Water channels could also be visualized (Figure 3J), as well as ECM, as exemplified by isolates of *C. haemulonii* (LIP*Ch*4), *C. duobushaemulonii* (LIP*Ch*6), and *C. haemulonii* var. *vulnera* (LIP*Ch*5) (Figure 4A,B,C, respectively).

Similarly, Silva et al. [45] demonstrated that *C. glabrata* biofilms are also composed only by yeasts, while *C. parapsilosis* sensu strictu and *C. tropicalis* biofilms characteristics vary depending on the strain used. Those authors observed that some *C. parapsilosis* strains formed biofilms containing both yeast and pseudohypha morphologies, while others presented yeast cells only, and these findings showed no relation with the isolation site of each strain [45]. The majority of *C. tropicalis* isolates displayed only yeast cells, but a small number of isolates showed hyphal formation, especially appearing as long filaments [45]. The biofilm formed by *C. auris*, which is phylogenetically closer to the *C. haemulonii* species complex, is predominantly composed by budding yeast cells and occasionally pseudohyphae [46]. *C. albicans* biofilms, on the other hand, are classically composed by a basal yeast cell polylayer and an upper region formed by hyphal forms [44].

### 3.4. ECM Composition

**Figure 5:**
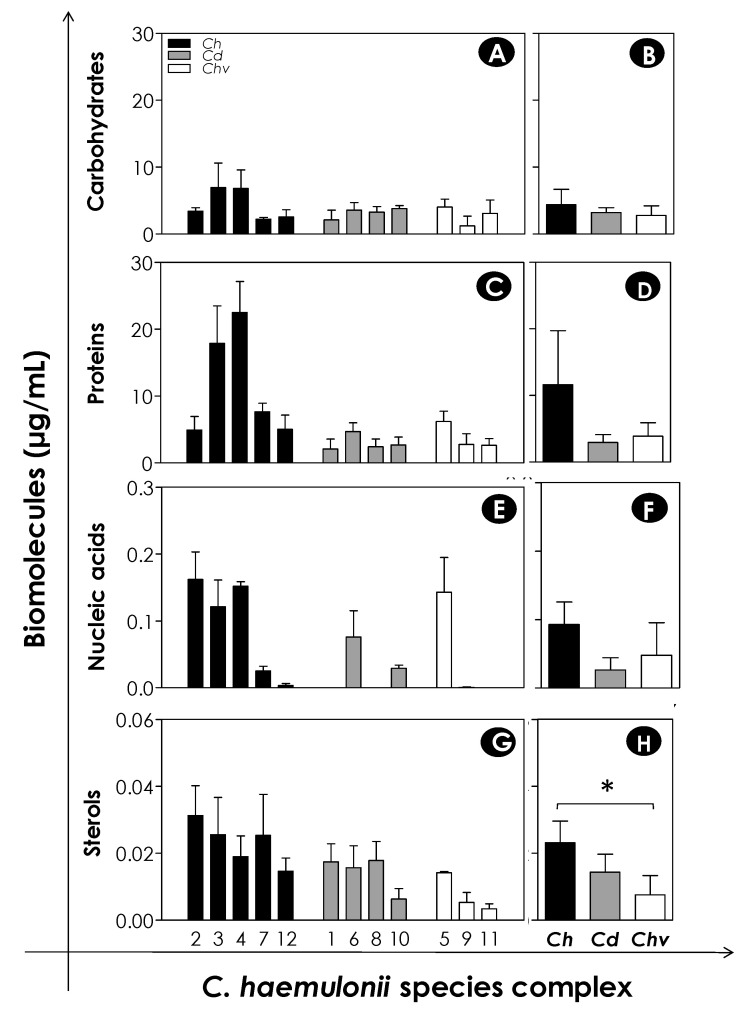
Main biomolecules forming the extracellular matrix (ECM) of the *C. haemulonii* species complex biofilms on polystyrene surface. Yeasts (200 μL containing 10^6^ cells) were placed to interact with polystyrene for 48 h at 37 °C. After that, ECM was extracted and carbohydrates, proteins, nucleic acids, and sterols were quantified as detailed in methodology section. The results were expressed as concentration (µg/mL) of each biomolecule per clinical isolate studied (**A**,**C**,**E**,**G**) and mean concentration per fungal species (**B**,**D**,**F**,**H**). The results are shown as mean ± standard deviation of three independent experiments. The symbol (*) indicates *p*-values < 0.05 (one-way ANOVA, Tukey’s multiple comparison test). The numbers on the X-axis of graphs represent each of the 12 clinical isolates of the *C. haemulonii* complex studied, in which *Ch* means *C. haemulonii*, *Cd* means *C. duobushaemulonii,* and *Chv* means *C. haemulonii* var. *vulnera*.

The ECM of biofilms from different *Candida* species exhibits a heterogeneous nature, which has already been thought to be associated to the roles of these components in biofilm architecture and dynamics [47]. The main components of ECM biofilms from *Candida* spp. are proteins, carbohydrates, lipids, and nucleic acids. Several studies have documented the participation of ECM biofilm in adhesion to surfaces, structural maintenance, defense against external aggressors, signaling, and enzymatic issues; however, the enhanced antimicrobial resistance is the most clinically important phenotype of biofilm mode of growth, which is of special concern in hospital settings [25,38,48]. Herein, we investigated the main classic components of the ECM of *Candida* spp. biofilms: proteins, carbohydrates, nucleic acids, and sterols. Among the evaluated components, proteins (mean of 11.61 ± 8.09 µg/mL for *C. haemulonii*, 2.97 ± 1.16 µg/mL for *C. duobushaemulonii,* and 3.88 ± 2.04 µg/mL for *C. haemulonii* var. *vulnera*) were found in greater quantity in the chemically extracted ECM from mature biofilms of all the clinical isolates, followed by carbohydrates (mean of 4.39 ± 2.30 µg/mL for *C. haemulonii*, 3.20 ± 0.74 µg/mL for *C. duobushaemulonii*, and 2.79 ± 1.42 µg/mL for *C. haemulonii* var. *vulnera*); nucleic acids (mean of 0.093 ± 0.074 µg/mL for *C. haemulonii*, 0.026 ± 0.035 µg/mL for *C. duobushaemulonii,* and 0.048 ± 0.082 µg/mL for *C. haemulonii* var. *vulnera*); and, lastly, sterols (mean of 0.023 ± 0.006 µg/mL for *C. haemulonii*, 0.014 ± 0.005 µg/mL for *C. duobushaemulonii,* and 0.007 ± 0.005 µg/mL for *C. haemulonii* var. *vulnera*) (Figure 5). Sterol amounts in *C. haemulonii* isolates were significantly higher when compared with those in *C. haemulonii* var. *vulnera* (*p* < 0.05; one-way ANOVA, Tukey’s multiple comparison test) (Figure 5).

Zarnowski et al. [47] described proteins and carbohydrates as the major components of *C. albicans* ECM biofilm, which included 458 distinct protein activities and three polysaccharides of functional importance (α-1,2 branched α-1,6-mannans associated with unbranched β-1,6-glucans forming a mannan-glucan complex, and β-1,3-glucans in a smaller part). Differences regarding non-*albicans Candida* species biofilms ECM composition were reported many years ago. In this sense, Silva et al. [45] documented that *C. parapsilosis* biofilm ECM exhibited high carbohydrate and low protein contents; on the other hand, *C. tropicalis* exhibited high contents of both carbohydrates and proteins, while *C. glabrata* showed low contents of both carbohydrates and proteins.

### 3.5. Biofilm Formation on Medical Devices

**Figure 6:**
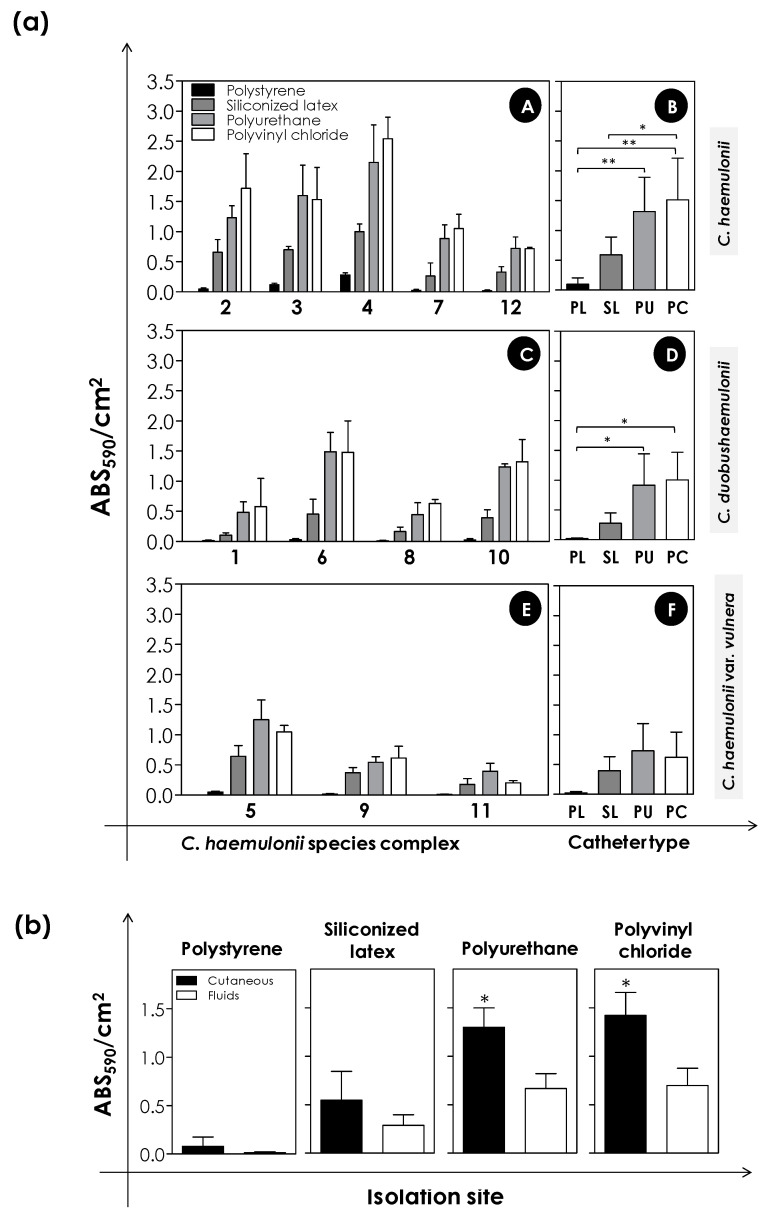
Biofilm formation on different catheter types by clinical isolates comprising the *C. haemulonii* complex. Fungal cells (200 μL containing 10^6^ cells) were placed to interact with polystyrene (PL) and different types of catheters (siliconized latex, SL; polyurethane, PU; and polyvinyl chloride, PC) for 48 h at 37 °C. Subsequently, the biofilm biomass was measured by the crystal violet incorporation (590 nm). (**a**) The results were expressed as ABS_590_/cm^2^ for each clinical isolate studied (A,C,E) and mean per each catheter type (B,D,F). Values represent the mean ± standard deviation of three independent experiments. The (*) indicates *p*-values < 0.05 and (**) *p*-values < 0.01 (one-way ANOVA, Tukey’s multiple comparison test). The numbers on the X-axis of the graphs represent each of the 12 clinical isolates of the *C. haemulonii* complex studied. (**b**) Comparison of biofilm biomass produced by the clinical isolates on polystyrene and each catheter type regarding the isolation sites (cutaneous and fluids). The 12 isolates were divided into two groups: cutaneous, including nail and skin (*n* = 7); and fluids, including blood, urine, and bronchoalveolar lavage (*n* = 5) (Table 1). (*) indicates *p*-values < 0.05 (unpaired Student’s *t*-test).

Catheter-related infections are considered a real problem in the medical arena around the world. Candidemia related to catheter use has already been reported in a variety of *Candida* species, including *C. haemulonii* species complex, resulting from the ability of this and other fungal pathogens to adhere to the catheter surface and, consequently, reach the bloodstream mainly of immunocompromised individuals [6]. For this reason, we decided to evaluate the *C. haemulonii* species complex biofilm formation capacity on the surface of different types of catheters currently used in the hospital environment—a latter bladder catheter made of siliconized latex, a nasoenteric catheter made of polyurethane, and a nasogastric catheter made of polyvinyl chloride. Biofilm formation on these materials was compared to that on polystyrene, a classical substratum used for biofilm analysis (Figure 1). The clinical isolates of the *C. haemulonii* complex were incubated for 48 h at 37 °C with the different materials and the biomass was measured by the incorporation of crystal violet. The results were expressed as absorbance (ABS_590_)/cm^2^, as the catheters have different dimensions. Our results stressed that the biofilm formation was significantly bigger over the different catheter types when compared with polystyrene regarding all the clinical isolates tested, demonstrating the risk that these clinical isolates would represent in the hospital settings, especially in individuals using nasogastric, nasoenteric, and urinary catheters (Figure 6(aA,aC,aE)). Additionally, biofilm formation on polyurethane and polyvinyl chloride catheters was comparable, with no significant differences between them (Figure 6(aA,aC,aE)). When comparing the mean biofilm formation per species of the *C. haemulonii* complex between the different substrates, we observed that the biofilms formed on polyurethane and polyvinyl chloride catheters were significantly bigger than on polystyrene for both *C. haemulonii* and *C. duobushaemulonii*. Further, biofilms formed on polyvinyl chloride catheters were significantly bigger than on siliconized latex only for *C. haemulonii* (Figure 6(aB,aD)), while for *C. haemulonii* var. *vulnera*, no differences were observed (Figure 6(aF)). Additionally, in relation to the isolation site, cutaneous (fungal isolates LIP*Ch*2, LIP*Ch*3, LIP*Ch*4, and LIP*Ch*7 of *C. haemulonii*; LIP*Ch*1 and LIP*Ch*6 of *C. duobushaemulonii*; and LIP*Ch*5 of *C. haemulonii* var. *vulnera*) versus fluids (fungal isolates LIP*Ch*12 of *C. haemulonii*; LIP*Ch*8 and LIP*Ch*10 of *C. duobushaemulonii*; and LIP*Ch*9 and LIP*Ch*11 of *C. haemulonii* var. *vulnera*) (Table 1), we observed that biofilm formation on the polyurethane (*p* = 0.0427, unpaired Student’s*t*-test) and polyvinyl chloride catheters (*p* = 0.0472, unpaired Student’s*t*-test) by the isolates from cases of cutaneous candidiasis was significantly higher when compared with isolates obtained from body fluids (Figure 6b). However, for polystyrene and siliconized latex catheters, no statistically significant differences (*p* > 0.05) were observed in this regard (Figure 6b).

Estivill et al. [49], for example, demonstrated the ability of different *Candida* species (*C. albicans*, *C. parapsilosis*, *C. tropicalis*, *C. glabrata*, and *C. krusei*) to form biofilm on different catheter types, and as observed in our clinical isolates, the biofilms formed on the polyurethane and polyvinyl chloride catheters presented very close values for all species studied. Additionally, our group has previously demonstrated the ability of filamentous fungi from *Scedosporium* spp. and *Lomentospora prolificans* to form biofilm on these same catheters [50].

The biofilm formation capacity of *Candida* spp., with a special focus on *C. albicans*, on medical devices has been extensively studied over time. Indeed, the nature of substratum used really influences the biofilm formation. For example, *C. albicans* form better biofilms on soft materials of dentures than on acrylic surfaces [51]. Similarly, *C. albicans* form better biofilms in silicone elastomer and latex surfaces in comparison with polyvinyl chloride and, on the other hand, construct weaker biofilms on polyurethane and silicone [52]. Interestingly, chemical changes made on the surface of medical devices can also interfere in *C. albicans* biofilm formation. For instance, a significant reduction in biomass and metabolic activity of *C. albicans* biofilm was detected when fungal cells were putted to adhere on polyetherurethane covered with 6% of polyethylene oxide [53]. Such differences should be considered, when possible, in the choice of biomaterials to minimize the development of catheter-related *Candida* infections.

## 4. Conclusions

Collectively, the present study demonstrated the ability of the *C. haemulonii* species complex to form biofilm on different types of inert substrates, which is an incontestable virulence attribute associated with catheter-related candidemia in hospitalized individuals, representing a serious problem especially when dealing with multidrug-resistant pathogens such as the *C. haemulonii* species complex. Additionally, our results provide new data about *C. haemulonii* species complex biofilm ECM composition.

## Figures and Tables

**Table 1:** Clinical isolates used in the present work.

Species Code (GenBank Acession Number)	Isolation Site
***Candida haemulonii***	
**LIP*Ch*2** (KJ476194)	Cutaneous (sole of the foot)
**LIP*Ch*3** (KJ476195)	Cutaneous (toe nail)
**LIP*Ch*4** (KJ476196)	Cutaneous (finger nail)
**LIP*Ch*7** (KJ476199)	Cutaneous (toe nail)
**LIP*Ch*12** (KJ476204)	Fluid (blood)
***Candida duobushaemulonii***	
**LIP*Ch*1** (KJ476193)	Cutaneous (finger nail)
**LIP*Ch*6** (KJ476198)	Cutaneous (toe nail)
**LIP*Ch*8** (KJ476200)	Fluid (blood)
**LIP*Ch*10** (KJ476202)	Fluid (bronchoalveolar lavage)
***Candida haemulonii* var. *vulnera***	
**LIP*Ch*5** (KJ476197)	Cutaneous (toe nail)
**LIP*Ch*9** (KJ476201)	Fluid (urine)
**LIP*Ch*11** (KJ476203)	Fluid (blood)
